# Cytokine MIF Enhances Blood-Brain Barrier Permeability: Impact for Therapy in Ischemic Stroke

**DOI:** 10.1038/s41598-017-16927-9

**Published:** 2018-01-15

**Authors:** Yu-Chuan Liu, Yung-Hsu Tsai, Sung-Chun Tang, Houng-Chi Liou, Kai-Hsiang Kang, Horng-Huei Liou, Jiann-Shing Jeng, Wen-Mei Fu

**Affiliations:** 1 Institute of Pharmacology, College of Medicine, National Taiwan University, Taipei, 10051 Taiwan; 2 Department of Neurology, National Taiwan University Hospital, Taipei, 10002 Taiwan

## Abstract

Ischemic stroke is a devastating disease with limited therapeutic options. It is very urgent to find a new target for drug development. Here we found that the blood level of MIF in ischemic stroke patients is upregulated. To figure out the pathological role of MIF in ischemic stroke, both *in vitro* and *in vivo* studies were conducted. For *in vitro* studies, primary cortical neuron cultures and adult rat brain endothelial cells (ARBECs) were subjected to oxygen-glucose deprivation (OGD)/reoxygenation. Middle cerebral artery occlusion (MCAo) rodent models were used for *in vivo* studies. The results show that MIF exerts no direct neuronal toxicity in primary culture but disrupts tight junction in ARBECs. Furthermore, administration of MIF following MCAo shows the deleterious influence on stroke-induced injury by destroying the tight junction of blood-brain barrier and increasing the infarct size. In contrast, administration of MIF antagonist ISO-1 has the profound neuroprotective effect. Our results demonstrate that MIF might be a good drug target for the therapy of stroke.

## Introduction

Stroke is a leading cause of death and long-term disability worldwide. WHO reported that about 15 million people have a stroke each year. With the aging population, these numbers are surly to increase greatly in the future. There are two main types of stroke: hemorrhagic stroke and ischemic stroke; among them, approximately 80 percent of strokes are ischemic^1^. Among numerous risk factors of stroke, hypertension is the major risk factor, leading to about 3-fold increase in the risk of stroke^2^ and poor outcome^3^ compared with normal blood pressure. Until now, intravenous tissue type plasminogen activator (IV-tPA) to restore blood flow in early phase remains the treatment of choice for reducing brain injury following stroke. However, the benefit of t-PA in acute ischemic stroke is limited by its narrow therapeutic time window and contraindications^4^. Thus, new therapeutic option for ischemic stroke is in urgent need.

The attenuation of blood flow to the brain will initiate the ischemic cascade, a complex sequence of event that ultimately leads to neuronal death, causing damage to the brain tissue and results in neurological deficits. Ischemic cascade includes the damage of blood-brain barrier (BBB), a highly selective barrier separating the brain from the circulatory system. The tight junctions between brain endothelial cells form the intercellular barrier. During ischemia, the degradation of tight junctions leads to the disruption of BBB, which directly contributes to the influx of immune cell and inflammatory materials, and also cerebral edema^5^. Inhibition of BBB disruption after stroke may reduce brain edema and neuronal damage. Post-ischemic inflammation also has important contribution on tissue injury. In stroke, the increase of circulating cytokines has been detected after cerebral ischemia in human and has been demonstrated to modulate the infract size and brain edema in rodent stroke models^6–9^.

Macrophage migration inhibitory factor (MIF) contains 114 amino acid with a molecular weight of 12.5-kDa and is expressed in a diversity of cell types, including T cells, macrophages, monocytes, endothelial cells^10^, and also in activated platelets^11^. It is recognized as a multifunctional cytokine participating in both immune and inflammatory responses. In addition, MIF is also found to possess pleiotropic functions, such as suppressing apoptosis, chemotactic responses^12^, wound repair^13^. The expression of MIF is upregulated in several diseases^14–16^. It has been reported that the activity of MIF promoter is significantly upregulated in response to hypoxia^16^, and MIF is dysregulated in rodent ischemic stroke model^15^.

Although cytokine MIF has been studied for decades, its pathological characters and the action mechanism in the brain remain far from clear. Here we will investigate the role of MIF in BBB permeability and neurologic impairment following acute ischemic stroke.

## Results

### MIF is upregulated in stroke patients

**Figure 1:**
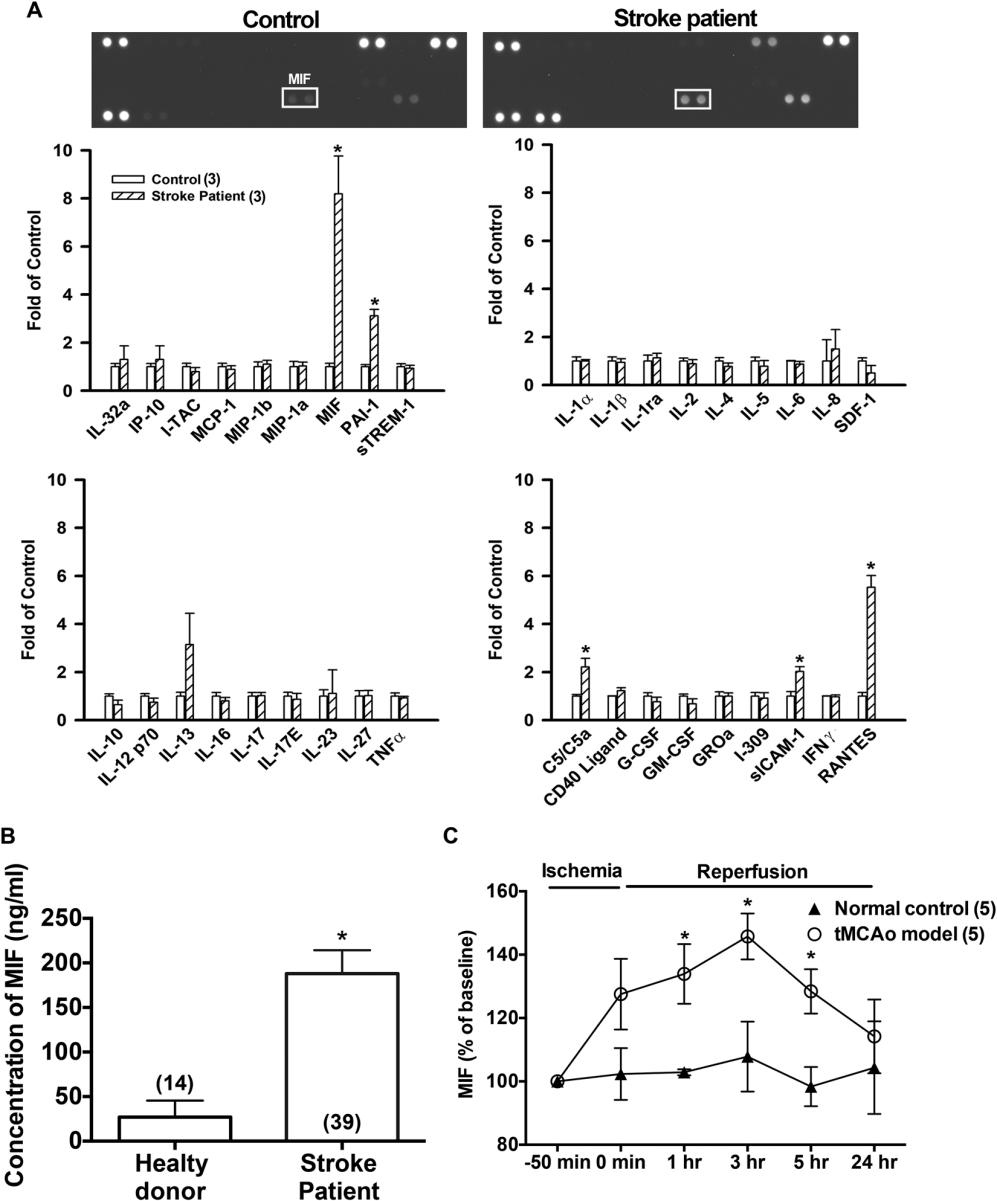
Blood concentration of MIF is elevated after ischemic stroke in patients and in rodent tMCAo model. (**A**) The blood samples were collected from healthy donors and ischemic stroke patients. The representative graphs and quantitative results of cytokine array (n = 3). (**B**) The plasma levels of MIF in ischemic stroke patients (n = 39) and in healthy donor (n = 14). Note that MIF was increased in patients before tPA treatment. (**C**) The serum level of MIF in tMCAo rat model was measured at different time points by using ELISA kits (n = 5). Data are shown as the mean ± S.E.M. (n). *p < 0.05 compared with normal control (healthy donors) or baseline level in rats.

Cytokines released from cells play an important role in many physiological and pathological processes. Traumatic brain injury, ischemic stroke, toxins, and many other diseases can affect the expression of cytokines^17–19^. Cytokine array was thus used to examine the profile of various cytokines/chemokines in the plasma of stroke patients. It was found that the plasma level of MIF was markedly elevated after stroke onset (n = 3, Fig. 1A). Since the level of MIF markedly increased, ELISA kits were then used to detect the concentrations of MIF in healthy donor and stroke patients before tPA administration. As shown in Fig. 1B, MIF was significantly increased in acute ischemic stroke patients (188.0 ± 26.3 ng/ml, n = 39) as compared with healthy donors (26.9 ± 5.0 ng/ml, n = 14).

### Upregulation of MIF in tMCAo rats

Male Wistar rats were performed by tMCAo for 50 minutes. Rat blood samples were collected before and after ischemia/reperfusion. The blood samples in normal control group were collected at different time points without tMCAo. The levels of MIF significantly elevated at earlier time intervals of 1~5 hours after ischemia/reperfusion (Fig. 1C), and the baseline value of MIF is 29.6 ± 2.1 ng/ml. These results indicate that the expression of MIF in peripheral blood was upregulated following ischemia in stroke patients as well as in experimental animal model.

### MIF leads to the disruption of tight junction in ARBECs

**Figure 2:**
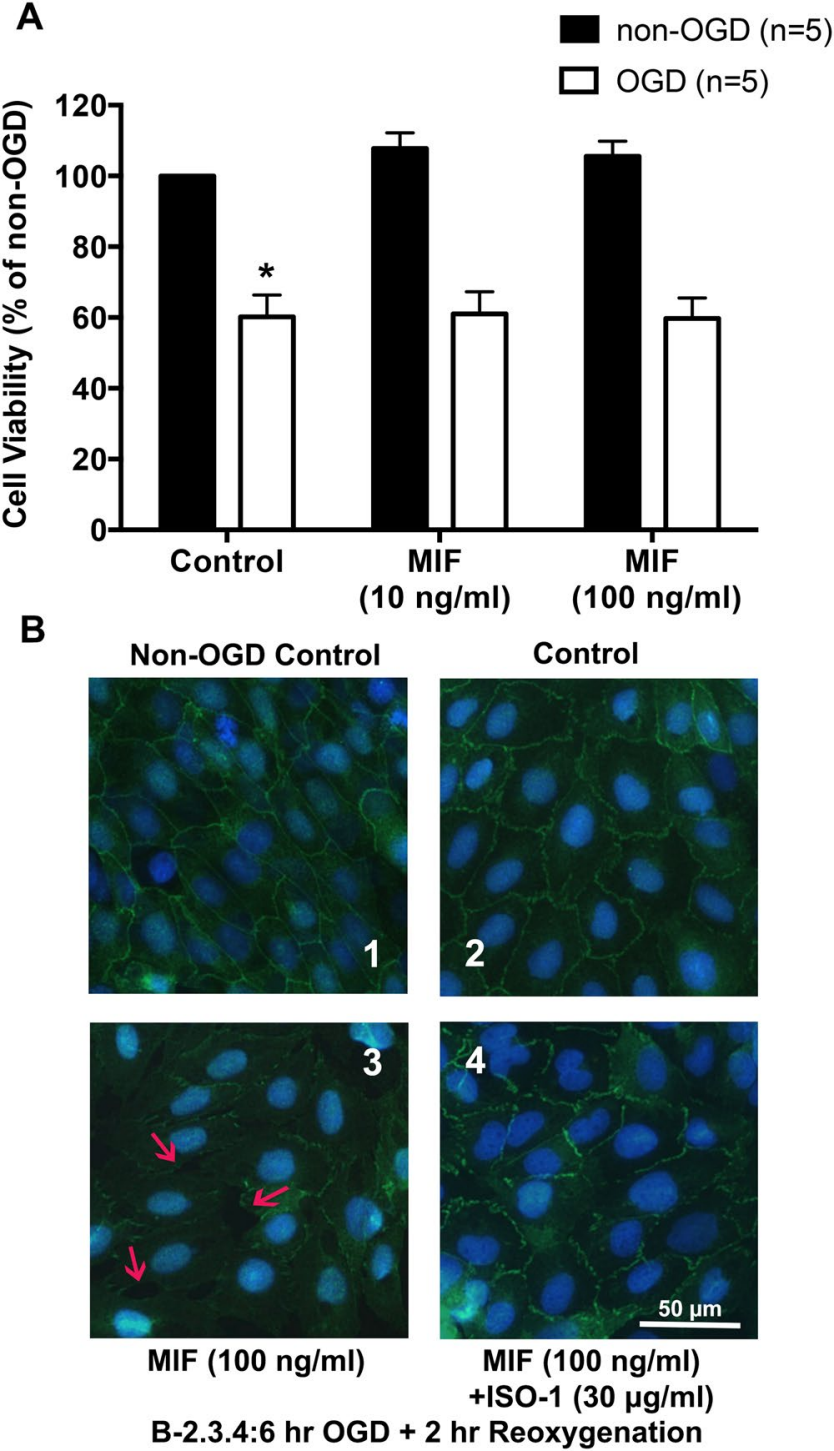
MIF disrupts tight junction protein in ARBECs but does not affect the neuronal viability in primary neuronal culture. (**A**) MIF (10 ng/ml or 100 ng/ml) was added into the primary cortical neuron culture right after 1.5 hours OGD and cultures were collected for analysis 16 hours later. Note that MIF treatment did not directly affect the neuronal viability. (**B**) ARBECs were exposed to oxygen glucose deprivation (OGD) for 6 hours and then reoxygenation for another 2 hours in pictures 2, 3 and 4. Note that OGD caused the disruption of ZO-1 (tight junction protein), which was aggravated by MIF treatment and inhibited by ISO-1 co-administration. Bars are means ± S.E.M. of 5 separate experiments run in triplicate. *p < 0.05 compared with control non-OGD group.

In order to examine the pathological role of MIF, we used the primary neuronal culture of cerebral cortex derived from Wistar rat embryos (E17). On Day-15, the cell culture was subjected to 1.5 hours OGD and followed by 16 hours reoxygenation. During reoxygenation, MIF (10 ng/ml and 100 ng/ml) were added to the culture. However, MTT assay revealed that MIF had no significant effect on the neuronal survival in both non-OGD and OGD groups (Fig. 2A).

Stroke causes the change of BBB integrity and results in injury response. We thus examined whether MIF affects the BBB permeability following OGD/reoxygenation. Adult rat brain endothelial cells (ARBECs) were exposed to OGD for 6 hours and followed by reoxygenation for 2 hours. During reoxygentation phase, cell culture was added MIF (100 ng/ml) with or without ISO-1 (30 µg/ml). Immunofluorescence staining revealed that OGD/reoxygenation caused the disruption of ZO-1, which was further exacerbated by MIF treatment (100 ng/ml). However, addition of MIF and ISO-1 in the cell culture simultaneously could antagonize the disruption effect on tight junction by MIF.

These results indicated that MIF did not directly affect the viability of neural cells; however, MIF led to the disruption of tight junction under OGD/reoxygenation in cell culture.

### MIF increases BBB permeability following tMCAo

**Figure 3:**
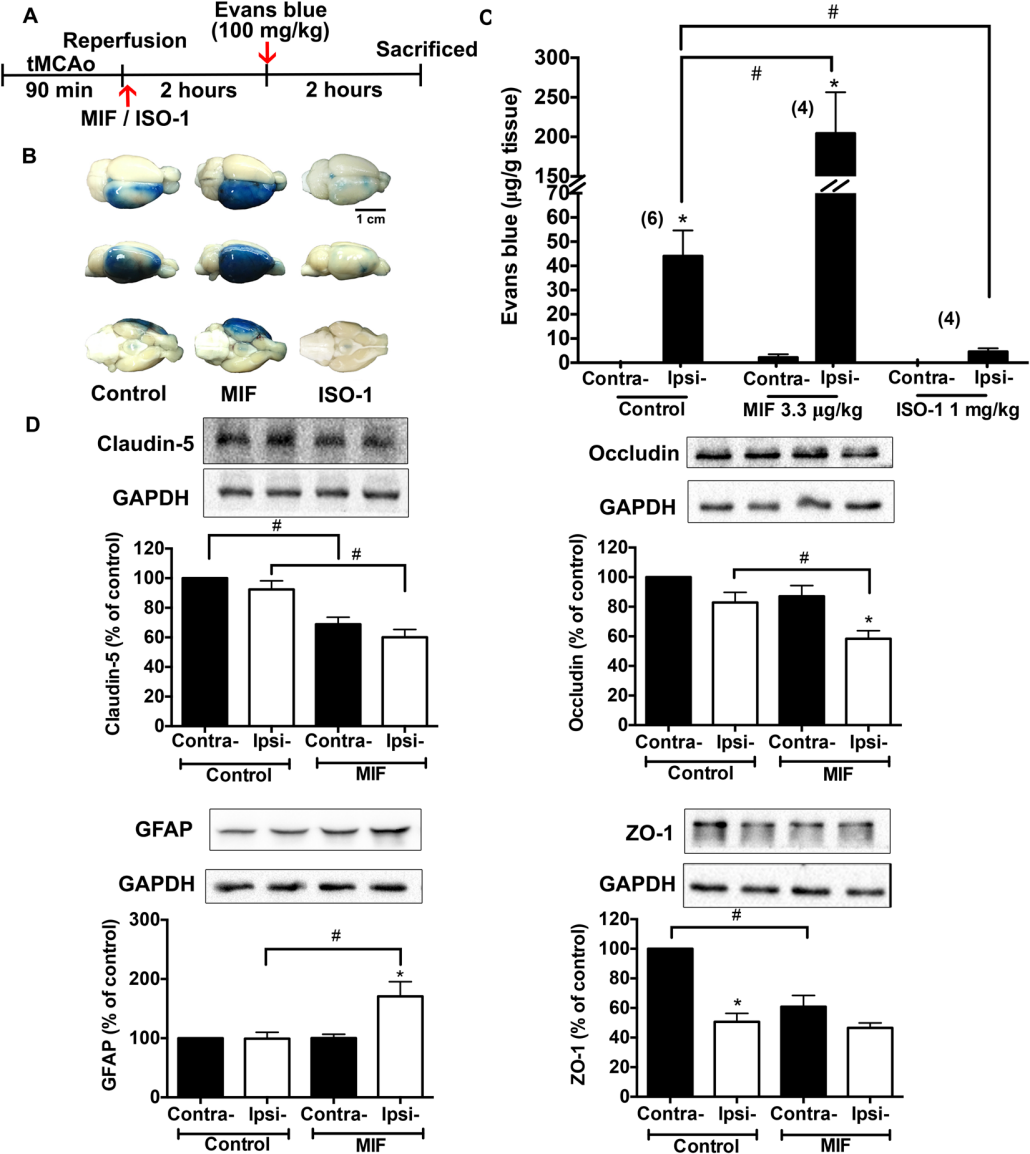
Administration of exogenous MIF increases and ISO-1 inhibits the BBB permeability following tMCAO in rats. Evans blue dye was used to evaluate the BBB permeability *in vivo*. (**A**) The time-course of BBB permeability assay. (**B**) Representative images of Evans blue dye in brain tissue. Note that Evans blue dye increased in ipsilateral cortex following tMCAo. MIF increased and ISO-1 inhibited stroke-induced increase of BBB permeability. (**C**) The summarized results of dye content in the cortex tissue. (**D**) The expressions of GFAP and tight junction proteins occludin, claudin-5 and ZO-1 in brain tissue (full-length blots are presented in Supplementary Figure 1). Data are presented as mean ± S.E.M. (n = 4–6 in Evans blue assay, and n = 5 in Western blotting). *p < 0.05 compared with contralateral cortex. ^#^*p* < 0.05 compared with control group.

In animal model, Evans blue assay was used to evaluate the BBB permeability. ISO-1 (4,5-Dihydro-3-(4-hydroxyphenyl)-5-isoxazoleacetic acid methyl ester), a potent and cell-permeable small molecule, inhibits the interaction between MIF and its receptor CD74^20^. After tMCAo for 90 minutes, rats were exogenously administered with MIF or ISO-1 from femoral vein right after reperfusion. Evans blue dye (100 mg/kg) was administered 2 hours later, and the permeability was evaluated further 2 hours later (Fig. 3A).

BBB leakage was dramatically increased in the ipsilateral hemisphere of MIF (3.3 µg/kg)-treated rats (204.3 ± 52.2 µg/g tissue) compared with the control group (44.1 ± 10.6 µg/g tissue). In contrast, ISO-1 (1 mg/kg) showed a protective action in BBB function (4.6 ± 1.4 µg/g tissue) (Fig. 3B and C). These results indicated that MIF increased the BBB permeability, whereas, ISO-1 inhibited the BBB leakage in ischemia/reperfusion animal models. Furthermore, the expression of occludin, claudin-5 and ZO-1 were decreased and the expression of GFAP (the marker of activated astrocytes^21^) was markedly elevated in the brain of MIF-treated rats 4 hours following tMCAo (Fig. 3D, n = 5).

### Exogenous administration of MIF increases, and ISO-1 decreases infarct volume in tMCAo rats

**Figure 4:**
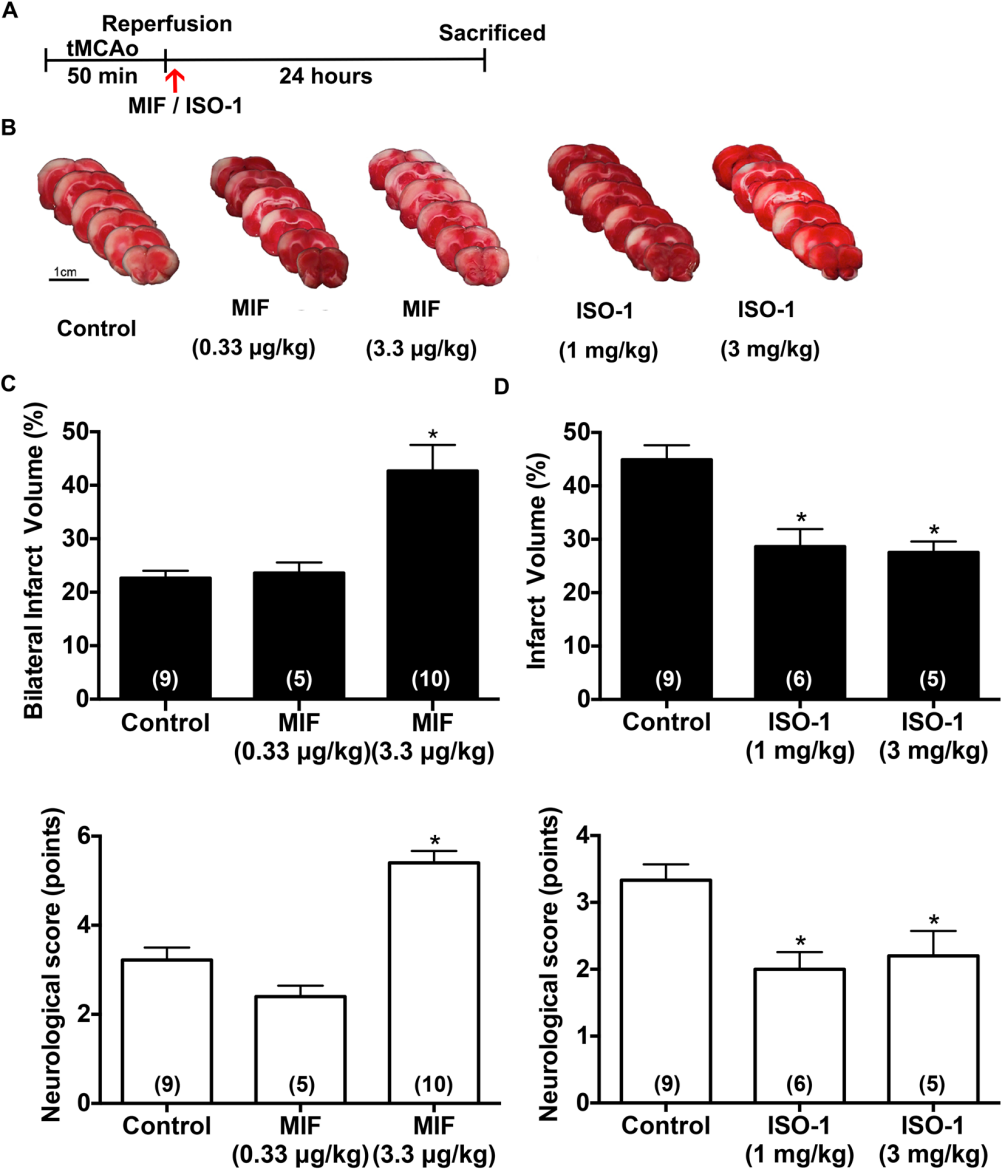
MIF increases and ISO-1 decreases the ischemia/reperfusion-induced infarction in tMCAo rat model. MIF at 0.33 μg/kg or 3.3 μg/kg was intravenously injected to rats right after tMCAo-reperfusion. The neurological scores and brain infarction of rats were evaluated 24 hours later. (**A**) The time-course of tMCAo and the administration of MIF and ISO-1. (**B**) TTC-stained sections at 24 hours after ischemia/reperfusion. Note that MIF (3.3 μg/kg) increased and ISO-1 decreased the infarct volume. (**C**) Summarized results of bilateral cortical lesions and neurological function were evaluated following MIF treatment. (**D**) Summarized results of the infarct volume (right cortical lesions only) and the neurological function following ISO-1 treatment. Data are presented as mean ± S.E.M. (n = 5–10). *p < 0.05 compared with control group.

We then examined the infarct volume of MIF in ischemic stroke model. After tMCAo, rats were exogenously administered with MIF or MIF antagonist ISO-1 from femoral vein right after reperfusion (Fig. 4A). The bilateral infarct volume significantly increased in MIF (3.3 µg/kg)-treated group as compared to the control group (Fig. 4B and C, 42.7 ± 4.9% vs. 22.6 ± 1.4%) 24 hours after stroke. In addition, MIF (3.3 µg/kg) group showed significant severe neurologic deficits (5.4 ± 0.3 points) compared with control group (Fig. 4C, 3.2 ± 0.3 points). Since three Wistar rats showed the bilateral damage in cortex following treatment with MIF (3.3 µg/kg), Fig. 4C is expressed as “Bilateral infarct volume (%)”. Furthermore, MIF at 10 µg/kg could cause the death of tMCAo rats (The mortality rate is 60%, n = 5).

It was also found that administration of ISO-1 at 1 mg/kg and 3 mg/kg right after tMCAo markedly decreased the infarct size to 28.6 ± 3.3% and 27.5 ± 0.9%, respectively as compared to control group (Fig. 4B and D, 44.9 ± 2.7%). In addition, ISO-1-treated group also showed better neurological function (Fig. 4D). These results demonstrated that MIF increased infarct volume with unfavorable neurological outcome and ISO-1 exerted neuroprotective action in ischemia/reperfusion rat model.

### Gliosis increases in the peri-infarct area of the ipsilateral hemisphere in MIF-treated rat

Astrocytes are the most abundant cell type in the brain and play essential roles in maintaining the normal function, cell communication, and also defense against oxidative stress^22^ in normal situation. Once the ischemic attack, astrocytes are activated. Activated astrocytes are believed to produce a variety of pro-inflammatory cytokines, and the high expression of cytokines can be detrimental to the severity of ischemic injury^23,24^. GFAP is the most important marker for the activated astrocytes^21^.

**Figure 5:**
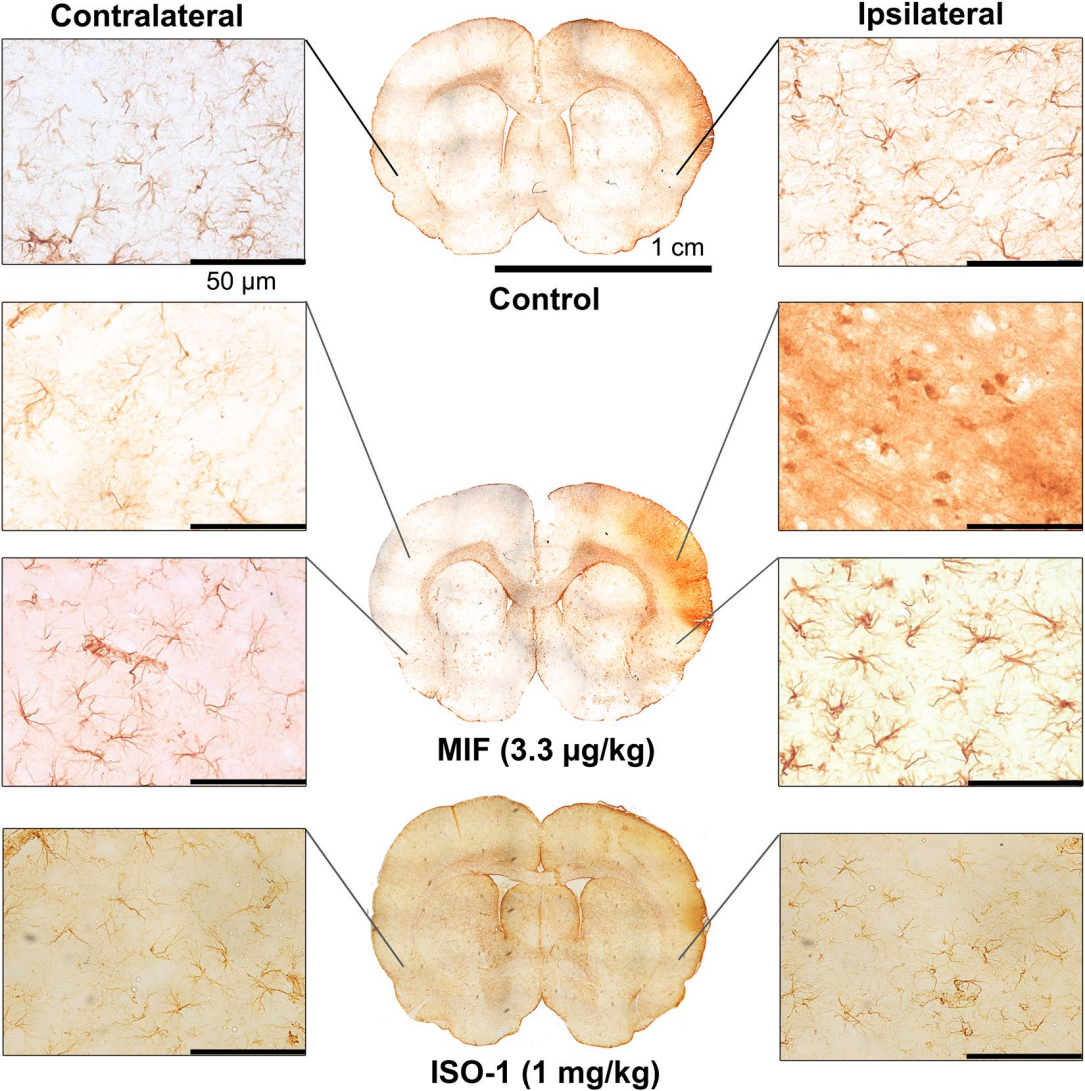
MIF treatment increases gliosis in the peri-infarct area of the ipsilateral hemisphere following tMCAo. The rat brain was collected 24 hours after ischemia/reperfusion. GFAP is a marker to identify the astrocytes in the cortex. Note that the expression level of GFAP in the ipsilateral peri-infarct region was markedly increased by the treatment of MIF (3.3 μg/kg) and decreased by ISO-1 (1 mg/kg) treatment.

GFAP-stained brain sections of tMCAO rats were shown in Fig. 5. Treatment with MIF (3.3 μg/kg) markedly increased the numbers of GFAP-positive cells 24 hours after tMCAO in peri-infarct area in ipsilateral side. The higher expression of activated astrocyte infers the severity of ischemic stroke in MIF-treated rats. In contrast, ISO-1-treated (1 mg/kg) rat showed the less GFAP-stained cell in the brain tissue after stroke.

### MIF exacerbates stroke injury in spontaneously hypertensive rat (SHR)

Hypertension is the most important risk factor for stroke. There are over 80% of acute stroke patients have poor control of their blood pressure^3^. According to the latest guidelines for early management of acute ischemic stroke patients from the American Heart Association (AHA)/American Stroke Association (ASA), tPA administration is not recommended in uncontrolled hypertension (over 185/110 mm Hg) patients^25^. Since the majority of stroke patients are presented with hypertension and are restricted to the use of tPA, we used SHR subjected to permanent ischemia model (pMCAo) to reflect the complicated clinical situation in patients with stroke.

**Figure 6:**
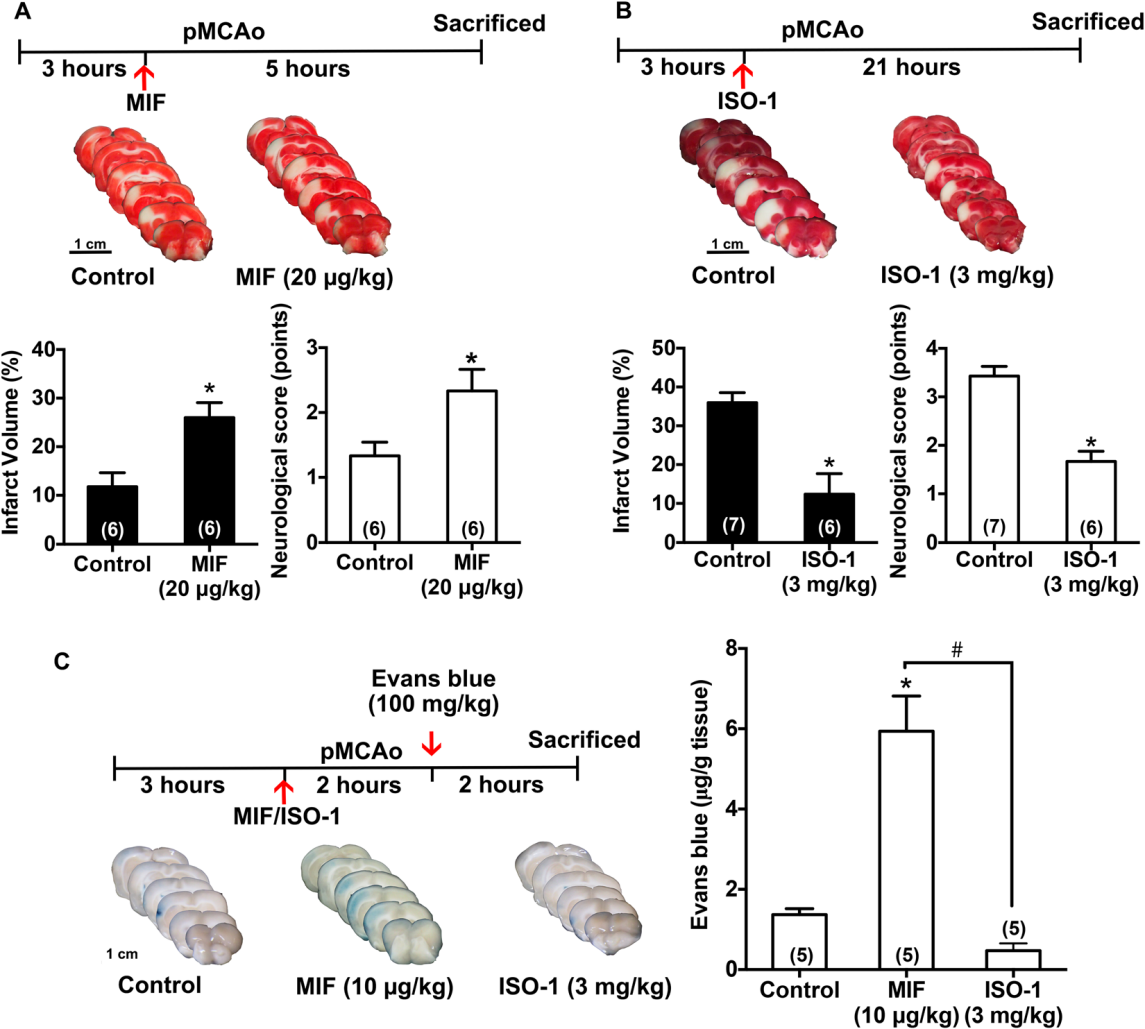
MIF exacerbates whereas ISO-1 ameliorates stroke injury in spontaneously hypertensive rat. Permanent cerebral ischemia was induced in SHR by occluding MCA alone (pMCAo). MIF or ISO-1 was injected 3 hours after pMCAo. Rats were then sacrificed 5 hours later in MIF experiments and 21 hours later in ISO-1 experiments. (**A**) Representative images, summarized results of infarct volume and neurological outcome by exogenous MIF (20 μg/kg, i.v.) administration. Note that MIF increased the infarct volume and neurological score. (**B**) Representative images, summarized results of infarct volume and neurological outcome by ISO-1 (3 mg/kg, s.c.) administration. Note that ISO-1 decreased the infarct volume and neurological score. The time-course of Evans blue dye assay was shown in the upper panel of (**C**). Representative images and quantitative results of Evans blue dye extravasation by exogenous MIF (10 μg/kg, i.v.) and ISO-1(3 mg/kg, i.v.) administration were shown in the lower and right panels, respectively. Note that MIF increased and ISO-1 decreased stroke-induced BBB permeability. Data are presented as mean ± S.E.M. (n = 5–7). *p < 0.05 compared with control group. ^#^*p* < 0.05 compared with MIF treatment group.

MIF (20 μg/kg) was administered 3 hours after pMCAo. In consistence with the phenomenon observed in Wistar rats, exogenous administration of MIF caused an increase in infarction 8 hours after pMCAo as compared to control group (Fig. 6A, 11.8 ± 2.9% vs. 26.0 ± 3.1%). However, ISO-1 reduced the lesion size at 24 hours (35.9 ± 2.6% vs. 12.4 ± 5.3%) and exhibited less severe neurologic deficits when given at 3 hours after pMCAo (Fig. 6B).

Further evaluation of cerebrovascular integrity also revealed that MIF-treated group (10 μg/kg, 3 hours post pMCAo) exhibited a marked increase of dye content in the extravascular compartment of the ischemic hemisphere as compared with control stroke animals, indicating an increase of BBB permeability among MIF-treated animals (Fig. 6C, 1.4 ± 0.2 vs 5.9 ± 0.9 µg/g tissue). The administration of ISO-1 (3 mg/kg, 3 hours post pMCAo) also demonstrated a decline in dye content as compared with control group (Fig. 6C, 1.4 ± 0.2 vs 0.5 ± 0.2 µg/g tissue). These results further indicate that MIF exerted an adverse impact on stroke injury induced by permanent cerebral ischemia under hypertension condition, particularly by aggravating the loss of cerebrovascular integrity after ischemic stroke; however, ISO-1 exerted protective role in ischemic stroke.

## Discussion

Stroke is a devastating disease characterized by neuronal damage and disability after alteration of blood flow in the brain. The pathological cascades after ischemia/reperfusion are complicated and still not fully elucidated. Compelling evidence suggests that pro-inflammatory cytokines and numerous chemokines are elevated in response to stroke, inducing the subsequent reactions, contributing to the post-stroke inflammation, and leading to neuronal damage^7^. To find out a possible drug target, we examined the profile of various cytokines/chemokines in the plasma of ischemic stroke patients and demonstrated that cytokine MIF was markedly elevated in the stroke patients. It has also been reported that the expression levels of MIF mRNA and protein^15,16,26^ are upregulated during acute phase of stroke, indicating that MIF is involved in the pathology of cerebral ischemia.

MIF has a pro-inflammatory action in local and systemic inflammatory responses^10^. Although evidences between the relationship of MIF and inflammation are mostly outside the brain, it still points out the possibility that MIF may critically affect the inflammatory reaction under pathological conditions^27–29^. Indeed, the implication of MIF in ischemic stroke has been revealed. The pathological role of MIF has been examined by Inácio *et al*.^14^ and shows that MIF knockout mice reduces neuronal death and promotes recovery of stroke-induced neurologic deficits following tMCAo^14^. The recent clinical research demonstrates that serum MIF level positively correlates to the severity of neurological function (by using NIHSS score) and also infarct volume in acute ischemic stroke patients^26^. In consistent with these studies, our findings indicated that MIF plays a pathological role in blood-brain barrier following stroke.

Blood-brain barrier provides a tight interface between the central nervous system and the peripheral circulation, maintaining the homeostasis of the microenvironment in the brain from harmful materials and pathogens^30^. Ischemic stroke, trauma, or other CNS disorders may cause the BBB leakage, permitting the infiltration of immune cells, inflammatory substances, pathogens into the brain. The disruption of BBB then causes the inflammatory reaction, aggravates brain edema, increases the intracranial pressure, and leads to the neuronal damage. The elevation of BBB permeability results in poor outcome after stroke has been well demonstrated^5^. In our study, MIF treatment destroyed the expression of tight junction in the BBB cell line (ARBECs) exposed to OGD/reoxygenation. Evans blue assay was further used to assess the BBB permeability and the Western blotting to examine the expression of tight junction proteins after stroke in rodent model. Here we found that BBB leakage was dramatically elevated and the expression of tight junction proteins-claudin-5, ZO-1, and occludin was decreased, by MIF treatment in ischemia/reperfusion rodent model. In contrast, MIF antagonist ISO-1 treatment decreased the Evans blue dye content in the brain tissue, indicating that ISO-1 exerts neuroprotective action in tMCAo by reducing BBB permeability.

MIF receptor expresses on endothelial cell and it has been reported that MIF promotes autophagy induction in endothelial cells, leading to dysfunction in endothelial barrier and the consequently increased vascular permeability^31^. These studies are consistent with our data from ARBECs cell line and *in vivo* permeability assay, indicating that MIF may exert its effect on BBB integrity directly. In addition, MIF regulates inflammatory response. Unlike most cytokines, MIF is constitutively expressed in intracellular space and rapidly released upon the exposition to stress. It is known that MIF promote the production of pro-inflammatory cytokines including TNFα, IL-1β and IL-6, which has been reported to increase BBB permeability^5,10^. Our data from stroke patient showed that the elevation of MIF is prior to other cytokines, suggesting its regulatory role in the inflammation of stroke. However, more evidence is needed to validate the precise mechanism of MIF in BBB disruption.

Astrocytes are known for maintaining the normal functions, such as structural support, neuronal metabolism, neurotransmitter synthesis, ion homeostasis, and the formation of blood-brain barrier^22,32^. The astrocytes become active, abnormal upregulation of GFAP and hypertrophy, and cause astrogliosis in response to ischemic damage^33^. In addition, the active astrocytes are capable of releasing inflammatory molecules, cytokines, chemokines, and nitric oxide, which are viewed to exacerbate neuronal injury^34,35^. Due to these properties, the activated astrocytes are considered to be destructive after ischemia. By Western blotting and the immunohistochemistry of GFAP, we found that MIF treatment obviously increased the gliosis in ipsilateral cortex in stroke model. Therefore, the higher expression of astrocyte may correlate with the severity of ischemic stroke in MIF-treated rats.

Further experimental studies were conducted to examine the infarct volume and the neurological deficits 24 hours after ischemia/reperfusion in rats. The results showed that MIF can significantly increase the infarct size, and worsen neurological behavior following ischemia/reperfusion injury. However, MIF did not exert the direct toxic effect on primary cortical neurons following OGD treatment. These results suggest that the enhancement of infarction by MIF is not derived from its direct neurotoxicity in CNS. On the other hand, administration of MIF antagonist ISO-1 not only reduced the infarct volume but also improved neuronal function, indicating the protective action of ISO-1.

Hypertension is the single most important modifiable risk factor for stroke. It has been reported that about 80% of stroke patients arrived at the emergency room with high blood pressure^3^. Additionally, restricted administration of tPA results in only a small number of patients receives thrombolysis, meaning that the majority of stroke patients resemble a permanent occlusion condition. The above statistics lead us to investigate the effect of MIF in pMCAo SHR, which could mimic the majority of stroke cases in a more clinically appropriate way. Interestingly, exacerbation of infarction and BBB disruption by MIF and the protective effect of ISO-1 were also demonstrated in SHR subjected to pMCAo. Although the pathophysiological course may vary between transient and permanent model due to the reperfusion injury as the extent of BBB disruption is increased by reperfusion^36^, the results showed that MIF also plays a destructive effect in permanent cerebral ischemia.

Previous studies have clarified that there are multiple strategies, such as reduction of ROS, deletion of harmful cytokines, administration of anti-apoptotic agents after stroke can effectively decrease the infarct volume in ischemic stroke^37^. Our results show a crucial character of MIF in mediating ischemic brain injury. MIF increases BBB permeability under ischemic attack. Furthermore, MIF exacerbates neuronal cell death and neurological deficits in the MCAo rodent model. On the other hand, administration of MIF antagonist produces protective effects against BBB permeability, infarction and neurological deficits following ischemic injury. Our study provides the pathological mechanism of MIF in ischemic stroke and MIF may be a good drug target for the therapy of the stroke.

## Materials and Methods

### Human blood sample

We included 39 patients and 14 healthy donors from National Taiwan University Hospital. The inclusion criteria for blood sample collection of stroke patients are patients (>20 year-old) diagnosed with acute ischemic stroke and eligible for rtPA injection. Those who are suspected with acute inflammation, have a contraindication to IV rtPA were excluded. Healthy subjects aged >20 year-old were included as control group. Blood samples were collected from ischemic stroke patients before tPA injection. For healthy subjects, blood samples were obtained at enrollment. The clinical research is approved by National Taiwan University Hospital Research Ethics Committee (201205113RIC), and participants provided written informed consent. All methods and procedures were performed in accordance with the regulation of Ministry of Health and Welfare in Taiwan regarding human research. The level of cytokines in plasma was examined using Human Cytokine Array Kit (R&D Systems, MN, USA). The blood concentrations of MIF were determined using human MIF ELISA kit (Raybiotech, GA, USA).

### Ischemic stroke model

Male Wistar rats and SHRs were purchased from BioLASCO Taiwan Co., Ltd. Rats were housed in groups of 3 per cage under a controlled 12-hour light/dark cycle (light from 08:00 to 20:00), at 23 ± 1 °C and 55 ± 5% humidity, with free access to food and water. Animal studies were approved by Institutional Animal Care and Use Committee of National Taiwan University (IACUC 20140026). All methods and procedures in this study were performed in accordance with the guidelines of the National Institutes of Health in the USA regarding laboratory animal welfare.

#### Wistar rats: Transient middle cerebral artery occlusion (tMCAo)

Male Wistar rats (280–310 g) were used. Transient MCAo were performed as described in supplementary. Briefly, two common carotid arteries and the right middle cerebral artery (MCA) were occluded for 50 minutes to produce ischemia. The rectal temperature of the rat was maintained at 37 °C by external warming. Rats were randomly assigned to the following groups: MIF (Prospec, NJ, USA) 0.33 µg/kg or 3.3 µg/kg, ISO-1 (Biovision, CA, USA) 1 mg/kg or 3 mg/kg or control groups with similar volume of sodium chloride 0.9%. The agent was injected from femoral vein right after blood reperfusion. The dosage of MIF and ISO-1 in animal studies was derived from the concentration in cell culture treatment.

#### Spontaneously hypertensive rats: Permanent Middle Cerebral Artery Occlusion (pMCAO)

13–15 week-old male spontaneously hypertensive rats (SHRs) with mean systolic pressures of 192.5 ± 2.8 mmHg were used. Permanent cerebral ischemia model was performed by occluding the right MCAO as described above with no reperfusion. Rats were randomly assigned to the following groups: MIF 20 µg/kg, ISO-1 3 mg/kg or control groups with similar volume of sodium chloride 0.9% (8 hours or 24 hours after pMCAO). The agent was injected from femoral vein (MIF) or by subcutaneous injection (ISO-1) 3 hours after pMCAO. The dosage of MIF and ISO-1 were titrated based on the concentration in cell culture treatment. The investigator conducting the following experiments was blind to the experimental groups.

### Measurement of blood MIF level in ischemic stroke animal model

The serum levels of MIF were measured in the Wistar rat using MIF ELISA kit (Cloud Clone Corp., TX, USA) according to the instructions. Rat blood was collected at several time points as follows: before tMCAo, right after ischemia/reperfusion of tMCAo, and at 1, 3, 5, 24 hours after reperfusion. The blood samples of normal control group were collected at different time points without the ischemic surgical procedure.

### Evaluation of blood-brain barrier permeability in ischemic stroke animal model

Wistar rats were anesthetized and operated for tMCAo for 90 minutes. MIF or ISO-1 was administered right after ischemia/reperfusion. The Evans blue dye (100 mg/kg; Sigma Aldrich, MO, USA) was injected into femoral vein 2 hours later. For SHR, MIF was administered 3 hours after ischemia and the dye was injected 5 hours after pMACo. Two hours after injection of Evans blue dye, rats were then perfused with saline until colorless perfusion fluid was obtained. The rat brains were removed, and right/left cortex was collected separately.

The samples were soaked in 2 ml of 50% trichloroacetic acid solution. After homogenization and centrifugation, the extracted Evans blue dye was diluted with ethanol (1:3), and fluorescence intensity was measured at 620 nm excitation and 680 nm emission wavelength by using fluorescence plate reader^38^. The tissue content of Evans blue dye was then quantified from a linear standard curve derived from known amounts of dye and was expressed as µg/g tissue.

### Determination of Infarct Volume by TTC Staining

Brain was isolated 24 hours after ischemia/reperfusion (8 hours for MIF-treated SHR) and six coronal sections with 2 mm thick were immersed in 2% solution of TTC (2,3,5-Triphenyltetrazolium chloride) at 37 °C for 10–15 min. Infarct volume was calculated using Image J. The investigators who were blind to experimental groups conducted the measurements and quantifications of infarct volume.

### Assessment of Neurologic Deficits

The neurological assessment was conducted by a blind observer 24 hour after MCAo. Table I in supplementary shows a modified set, which is based on mNSS. The assessment was graded on a scale of 0 to 10 (normal score, 0; maximal deficit score, 10) through the observation of motor, sensory, and also reflex function. One point is awarded for the inability to perform the test or for the lack of a tested reflex. A higher score indicates a more severe injury after stroke.

### Immunohistochemistry

The brain sections (30 μm-thick) were soaked in 3% H_2_O_2_ with 20% methanol in PBS for 30 minutes to block endogenous peroxidase activity. After blocking in 4% milk solution for 1 hour, slices were incubated with primary antibody against GFAP (1:500; Cell Signaling Technology, MA, USA) overnight at 4 °C and then with biotinylated secondary antibodies for 1 hour. Staining was revealed by the avidin-biotin complex system (Vector Laboratories, CA, USA) and developed by using 0.5 mg/ml diaminobenzidine and 0.01% H_2_O_2_ in TBS to produce brown reaction product.

### Oxygen-glucose deprivation (OGD) of cell culture

Immortalized adult rat brain endothelial cells (ARBECs) and primary cortex neuronal cells were used for *in vitro* assay. To apply OGD, the normal glucose medium in the confluent monolayer cell culture was replaced by serum-free Dulbecco’s Modified Eagle’s Medium without glucose, and then placed in hypoxia chamber (95% N_2_ and 5% CO_2_) at 37 °C for several hours. In non-OGD groups, the cell cultures with normal glucose medium were incubated in normoxia incubator (95% air, 5% CO_2_).

After OGD, the cell culture was changed back to the normal glucose medium with or without treatment (MIF and/or ISO-1), and further transferred back to regular normoxia incubator (95% air, 5% CO_2_) for another few hours. The concentration of MIF used was similar to the study by Zis *et al*. (2015).

### Immunofluorescence

ARBECs were seeded onto glass coverslips coated with type I rat tail collagen. After treatment, cells were fixed with 4% paraformaldehyde for 15 min and washed with PBS. After blocking with 4% BSA overnight at 4 °C, glass coverslips were incubated with rabbit antibodies against ZO-1 (1:100; Invitrogen, CA, USA) at 4 °C for a day. Following brief wash with PBS, glass coverslips were incubated with donkey anti-rabbit FITC-conjugated secondary antibody (1:200; Invitrogen, CA, USA) for 1 h. Finally, the glass coverslips were washed again, and then socked in DAPI solution (0.5 ug/ml) for 15 minutes. The coverslips were then mounted and visualized with fluorescence microscope.

### Western blotting

The expression levels of occludin, ZO-1, claudin-5, GFAP were determined by Western blotting. Primary antibodies: occludin (1:1000; Santa Cruz Biotechnology, CA, USA), claudin-5 (1:500, Aviva systems Biology, CA, USA), ZO-1 (1:1000; Invitrogen, CA, USA), GAPDH (1:10000; Santa Cruz Biotechnology, CA, USA), GFAP (1:1000; Santa Cruz Biotechnology, CA, USA).

### Statistic analysis

All data were expressed as Mean ± SEM. The difference between two groups was determined by Student’s t-test. The difference among groups was compared with one-way analysis of variance (ANOVA) followed by Tukey’s multiple-comparison test. Significance was defined as *p* < 0.05.

### Clinical Research Registration

The clinical research in this study is approved by National Taiwan University Hospital Research Ethics Committee (201205113RIC).

## Electronic supplementary material

Supplementary Information
