# Supplementary material for: Cytokine MIF Enhances Blood-Brain Barrier Permeability: Impact for Therapy in Ischemic Stroke
Source: Sci Rep. 2018 Jan 15;8:743. doi: 10.1038/s41598-017-16927-9 (PMC5768806; doi:10.1038/s41598-017-16927-9)
Supplement: Supplementary file 1 — Supplementary Information [file 41598_2017_16927_MOESM1_ESM.pdf]

Supplementary Information

Cytokine MIF Enhances Blood-Brain Barrier Permeability: Impact for Therapy in Ischemic Stroke

Yu-Chuan Liu[1†]; Yung-Hsu Tsai[1†]; Sung-Chun Tang[2]; Houng-Chi Liou[1]; Kai-Hsiang Kang[1]; Horng-Huei Liou[1]; Jiann-Shing Jeng[2*], Wen-Mei Fu[1*]

[1] Institute of Pharmacology, College of Medicine, National Taiwan University, Taipei, 10051, Taiwan.

[2] Department of Neurology, National Taiwan University Hospital, Taipei, 10002, Taiwan.

†These authors contributed equally to this work.

*Correspondence to Wen-Mei Fu, PhD, Institute of Pharmacology, College of Medicine, National Taiwan University, Taipei, 10051, Taiwan. Tel: 886-2-23123456#88319 Fax: 886-2-23417930 E-mail: wenmei@ntu.edu.tw and Jiann-Shing Jeng, MD, PhD, Department of Neurology, National Taiwan University Hospital, Taipei, 10002,Taiwan. Tel: 886-2-23123456#62143 Fax: 886-2- 2341-8395 E-mail: jsjeng@ntu.edu.tw

Supplemental Methods

Ischemic stroke model in Wistar rats

The rats were anesthetized by exposure to 1 to 3% isoflurane. Two common carotid arteries were exposed by anterior neck cervical incision and occluded by micro-artery clips. Under an operating microscope, a piece of skull was removed, the dura was opened with fine forceps, and the right middle cerebral artery (MCA) was tied with a 10-0 polyamide monofilament non-absorbable suture to produce ischemia. Both common carotid arteries and the right middle cerebral artery were occluded 50 minutes. After removing the artery clips and untying the suture, the blood flow was recovered. The rectal temperature of the rat was maintained at 37°C by external warming. Respiratory rate, heart rate and temperature were monitored during and after surgery. Animals were randomized to control group or treatment group using http://randomized.com/ .

Exclusion criteria of stroke rats

A total of 68 Wistar rats were submitted to tMCAo. Animals with inappropriate occlusion of the MCA (n=3), spontaneous reperfusion during occlusion (n=0), unable to regain blood flow (reperfusion) after removing the artery clips and untying the suture (n=1) or sudden death within 24 hours (n=5) were excluded from further analysis.

A total of 38 SHR were submitted to pMCAo. Animals with inappropriate occlusion of the MCA (n=3) were excluded from further analysis.

Cell culture of ARBEC

Immortalized adult rat brain endothelial cell (ARBECs) were seeded onto 100 mm dishes coated with type I rat tail collagen and maintained in M199 (Invitrogen, CA, USA) containing 1% D-glucose solution, 1% Eagle's basal medium amino acid solution (Gibco, NY, USA), 1% MEM vitamin solution (Gibco, NY, USA), 100 U/ml penicillin, 100 mg/ml streptomycin, 10% fetal bovine serum at 37°C in a humidified incubator under 5% CO_2 and 95% air. The growth medium was renewed every two days, and confluent cultures were passaged by trypsinization.

Primary cortex neuronal cultures

Cortex dissected from Wistar rat embryos (E17) was put into HBSS without calcium and magnesium (Gibco, Grand Island, NY, USA) followed by mechanical trituration and the dissociated cells were centrifuged at 10,000 r.p.m. for 3 minutes. The supernatant was removed, and the cells were resuspended in Neurobasal medium supplemented with B27

supplement (Invitrogen, CA, USA), 100U/ml penicillin and 0.1mg/ml streptomycin. The cells were incubated in a humidified incubator at 37°C, 5% CO_2 of for 14 days. Half of the medium was replaced every two days.

Supplementary Figure 1: The expressions of GFAP and tight junction proteins occludin, claudin-5 and ZO-1 in brain tissue (full-length blots)

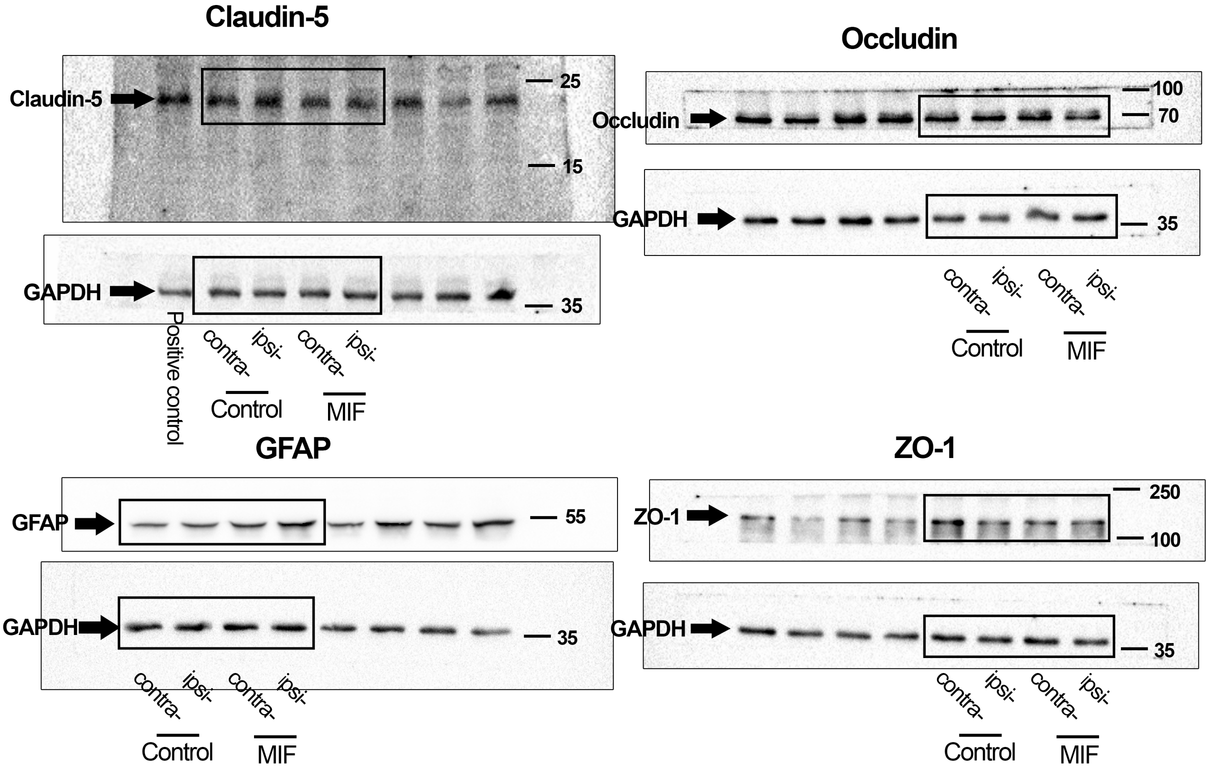

Supplemental Tables

Table I. The Assessment of Neurologic Deficits

**Motor tests**	**6**
Raising rat by tail	
Flexion of forelimb	1
Flexion of hindlimb	1
Head moved >10° to vertical axis within 30s	1
Placing rat on floor (normal=0; maximum=3)	
Normal walk	0
Inability to walk straight	1
Circling toward paretic side	2
Falls down to paretic side	3
**Reflex absence and abnormal movements**	**4**
Pinna reflex<br>(head shake when auditory meatus is touched)	1
Corneal reflex<br>(eye blink when cornea is lightly touched with cotton)	1
Startle reflex<br>(motor response to a brief noise)	1
Seizure, myoclonus, myodystony	1
**Maximum points**	**10**
**One point is awarded for inability to perform the tasks or for lack of a tested reflex: 7-10,severe injury; 4-6,moderate injury; 0-3, mild injury**	
